# Supplementary material for: Parental Self-Efficacy as a Predictor of Children’s Nutrition and the Potential Mediator Effect between the Health Promotion Program “Join the Healthy Boat” and Children’s Nutrition
Source: Int J Environ Res Public Health. 2020 Dec 17;17(24):9463. doi: 10.3390/ijerph17249463 (PMC7766743; doi:10.3390/ijerph17249463)
Supplement: Supplementary file 1 [file ijerph-17-09463-s001.pdf]

Table S1. Scales for parental self-efficacy on children's fruit and vegetable intake as well as consumption of sugar sweetened beverages

„I manage to make sure that my child eats fruits and vegetables daily, even if …"							
	Strongly disagree						Strongly agree
… my child rather wants to eat sweets or Fast Food….	☐	☐	☐	☐	☐	☐	☐
… we are eating outside…………………………………	☐	☐	☐	☐	☐	☐	☐
… it does not taste nice for my child………..………	☐	☐	☐	☐	☐	☐	☐
… I am too tired to prepare or arrange something…..	☐	☐	☐	☐	☐	☐	☐
… we are on vacation…………………………………...	☐	☐	☐	☐	☐	☐	☐
… there is not a wide variety in the supermarket..….	☐	☐	☐	☐	☐	☐	☐
„I manage to make sure that my child does not drink so many sugar sweetened beverages, even if…"							
	Strongly disagree						Strongly agree
… my child likes the drinks very much……..……….	☐	☐	☐	☐	☐	☐	☐
… we are on a birthday party or on a visit………...	☐	☐	☐	☐	☐	☐	☐
… we are eating outside……………………………..….	☐	☐	☐	☐	☐	☐	☐
… the drinks are available at home……………………	☐	☐	☐	☐	☐	☐	☐
… friends are drinking those drinks…………………..	☐	☐	☐	☐	☐	☐	☐
… my child has a lot of appetite for it……….………	☐	☐	☐	☐	☐	☐	☐

Table S2. Scales for parental outcome expectancies on children's fruit and vegetable intake as well as consumption of sugar sweetened beverages

„If my child eats fruit and vegetables daily, then … "							
	Strongly disagree						Strongly agree
… it is a good protection against illnesses……………	☐	☐	☐	☐	☐	☐	☐
… my child has more energy……………...…………...	☐	☐	☐	☐	☐	☐	☐
… it takes a lot of time, because I need to prepare and arrange something…………………………….……..	☐	☐	☐	☐	☐	☐	☐
… it gives me a good feeling…………………………...	☐	☐	☐	☐	☐	☐	☐
… it takes more money while grocery shopping…….	☐	☐	☐	☐	☐	☐	☐
… my child is a role model for other children……….	☐	☐	☐	☐	☐	☐	☐

Cont. Supplementary Table S2. Scales for parental outcome expectancies on children's fruit and vegetable intake as well as consumption of sugar sweetened beverages

„If my child drinks less sugary sweetened beverages, then …"							
	Strongly disagree						Strongly agree
… it is good for its teeth……………………………..….	☐	☐	☐	☐	☐	☐	☐
… it is good for its bodyweight………………………..	☐	☐	☐	☐	☐	☐	☐
… my child drinks less than it should…...……………	☐	☐	☐	☐	☐	☐	☐
… it takes a lot of time, because I need to provide something else……………………...……………	☐	☐	☐	☐	☐	☐	☐
… it gives me a good feeling…………………….…...	☐	☐	☐	☐	☐	☐	☐
… my child is a good role model for other children…	☐	☐	☐	☐	☐	☐	☐
